# Co-creating the EndoWatch - a smart wearable device for the management of hypothalamic dysfunction in pediatric brain tumor survivors: a pilot study

**DOI:** 10.3389/fendo.2026.1963615

**Published:** 2026-10-23

**Authors:** Sanne C. Hulsmann, Sarah B. Petras, Philippe Fraboulet, Yuan Lu, Johan P. de Graaf, Willemijn Plieger, Herke Jan Noordmans, Wim J.E. Tissing, Hanneke M. van Santen

**Affiliations:** 1 Department of Neuro-Oncology, Princess Máxima Center for Pediatric Oncology, Utrecht, Netherlands; 2 Department of Pediatric Endocrinology, University Medical Center Utrecht, Wilhelmina Children’s Hospital, Utrecht, Netherlands; 3 Corsano Health B.V., The Hague, Netherlands; 4 Department of Industrial Design, Eindhoven University of Technology, Eindhoven, Netherlands; 5 De Nederlandse Hypofyse Stichting (Dutch Pituitary Foundation), Nijkerk, Netherlands; 6 Department of Endocrinology, Leiden University Medical Center, Leiden, Netherlands; 7 EU Reference Network Rare Endocrine Conditions (Endo-ERN), Amsterdam, Netherlands; 8 Vereniging Kinderkanker Nederland (VKKN), De Bilt, Netherlands; 9 Department of Clinical Physics, University Medical Center Utrecht, Utrecht, Netherlands

**Keywords:** co-creating, craniopharyngioma, health monitoring, hypothalamic-pituitary dysfunction, late effects, low grade glioma, pediatric brain tumor, smart wearable

## Abstract

**Introduction:**

Hypothalamic dysfunction (HD) after treatment for a pediatric (supra)sellar tumor severely affects quality of life of patients, as well as their parents and families. Patients may develop signs of fatigue as a result of persistent disturbances in thermoregulation, sleep-wake rhythm, or hormonal insufficiencies. The management of HD requires around-the-clock care by the parent or caregiver. To support patients with HD and their parents/caregivers, we co-created a smart wearable device for continuous monitoring (EndoWatch) to detect early signs of hypothalamic dysregulation. We aimed to evaluate adherence to and user experiences of the first EndoWatch design.

**Methods:**

In co-creation with clinicians, patient representatives, and design researchers and engineers, an existing smart device (wearable sensor and mobile application (app)) was adapted to a first design tailored for this purpose. In a prospective, observational study, 10 pediatric brain tumor survivors with HD (8 children; 2 adults) tested the first design of the device for 14 days. The primary outcome was adherence to wearing the EndoWatch. Secondary outcomes included usability, learnability, and desirability (through questionnaires), and barriers and facilitators (through semi-structured interviews).

**Results:**

Ten patients participated (8 children, mean age 10.8 (SD 3.5); 2 adults, aged 18.9 and 31.0 years). Median overall adherence was 99.4% (IQR 22.8); two patients discontinued early due to wristband-related discomfort. Key facilitators were ease of use of the device and reliable technical performance. Main barriers included the absence of a separate caregiver app and limitations of the patient diary. These findings informed design modifications for future improvements of the device.

**Discussion:**

The concept of the first EndoWatch design was considered acceptable and usable by patients with HD and their caregivers. This study identified key barriers and the modifications required for improvement, which were incorporated into a second EndoWatch design, to be evaluated in a larger prospective study.

## 1 Introduction

Children and young adults with a suprasellar low-grade brain tumor, such as adamantinomatous craniopharyngioma (ACP) or suprasellar low-grade glioma (LGG), often have excellent survival rates. Quality of life, however, is in many cases compromised due to the sequelae of hypothalamic dysfunction (HD) caused by the tumor and/or its treatment (1–3). HD can severely impact daily life due to consequences of continuous imbalances in thermoregulation, sleep-wake rhythms, emotional behavior and impulse control, as well as consequences of (pan)hypopituitarism, such as central adrenal insufficiency and serum sodium fluctuations in patients with arginine vasopressin deficiency (AVP-D) (1, 4–6). These imbalances, when not adequately recognized or corrected can severely limit daily life as it can result in chronic fatigue, daytime sleepiness, inactivity, recurrent headaches or even seizures (3, 7–9). Delayed treatment can even be life-threatening in the case of inadequate or delayed management of an adrenal crisis in patients with central adrenal insufficiency (10, 11). In addition, HD can result in the development of hypothalamic obesity (HO), due to insatiable appetite (hyperphagia) and decreased energy expenditure (2, 12–14). Chronic fatigue, visual impairment (as a consequence of tumor-related damage to the optic chiasm), and loss of initiative may all lead to inactivity, and may further contribute to the development of (morbid) obesity. HO and fatigue are recognized as the most debilitating outcomes of HD by patients and their parents (9, 15). The burden of these sequelae, such as chronic fatigue and physical and psychological changes, may lead to symptoms of depression. Patients may become more isolated from their peers and often experience a decline in their level of school education due to physical or emotional complaints (16–18).

Management of HD should aim to maintain an optimal balance, striving for homeostasis, and may encompass continuous monitoring of body temperature, ensuring regular sleep patterns, promoting physical activity, and counseling for timely and adequate adjustment of hormone replacement therapy (19, 20). Caregivers of patients with HD bear the responsibility at home to maintain this balance, necessitating continuous, around-the-clock care, which may severely impact their daily life. They, in fact, function as the “external hypothalamus” of their child. For example, in case of adrenocorticotropic hormone (ACTH) deficiency, they are instructed to timely increase hydrocortisone stress dosages in case of increased physical (ie, fever) or emotional (ie, hospital visit) stress to avoid an adrenal crisis (11), and in case of AVP-D and adipsia, caregivers are counseled on how to monitor fluid balance on a daily basis (21). Patients with severe thermodysregulation receive frequent temperature checks at home to avoid (prolonged) hypo- or hyperthermia (6) (**Figure 1**).

**Figure 1:**
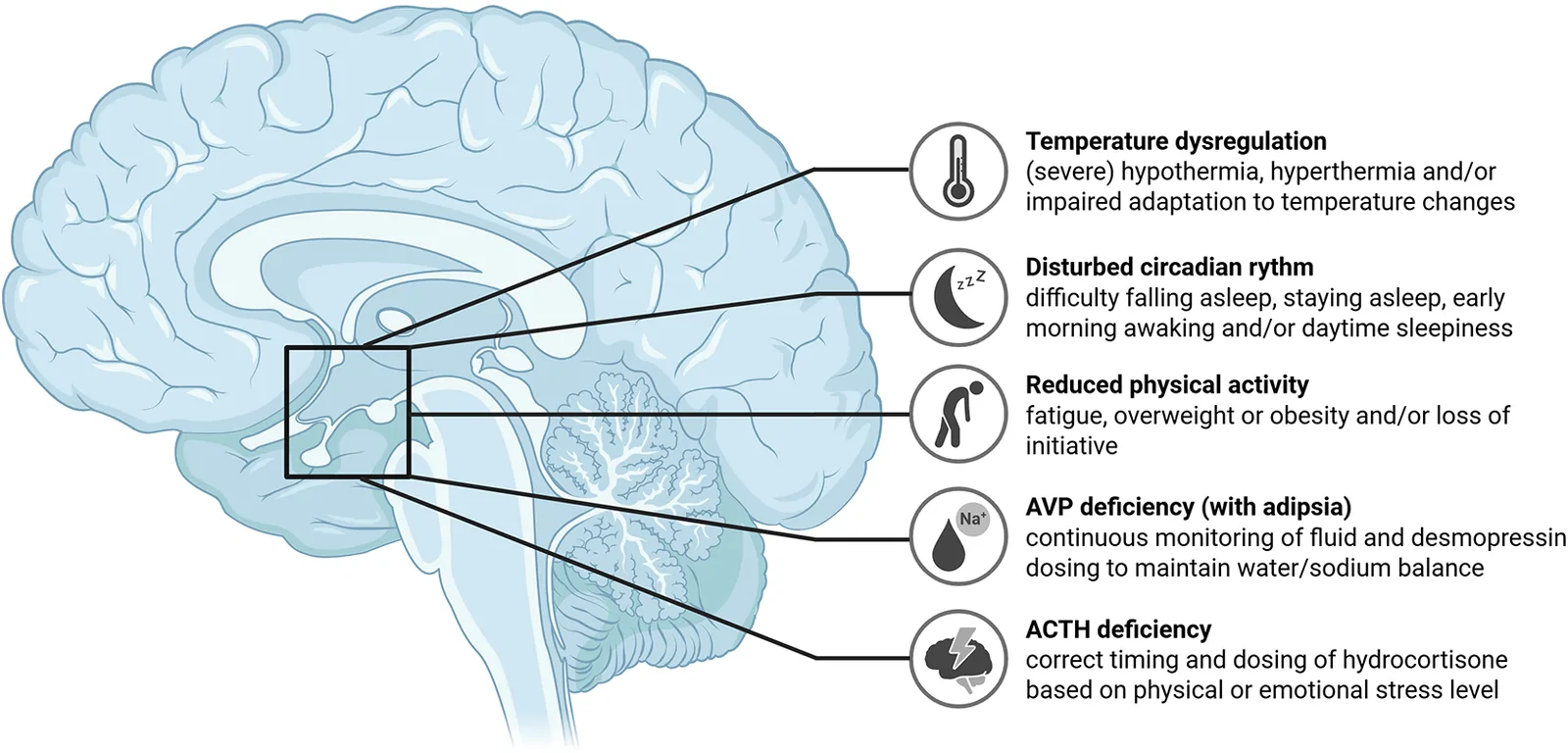
The imbalances caused by hypothalamic dysfunction (HD) that require 24/7 care and could be monitored by the EndoWatch. Created in BioRender. Hulsmann, S. (2026) https://BioRender.com/tszza6l.

Recognizing and managing HD is challenging due to its interindividual differences in clinical manifestations (4, 19). Currently, no one-size-fits-all treatment is available for patients with HD (2, 5). If certain hypothalamic imbalances were to be sensed earlier, more timely actions could be undertaken. For example, the severity of temperature fluctuations could be reduced if incline or decline of body temperature could be recognized earlier via continuous monitoring by a smart wearable, and more insight into sleep quality and sleep-wake times could be gained by sleep tracking. Monitoring of daily activity by tracking steps could provide insight into and promote physical activity. Potentially, recognizing physical or emotional stress, for example by variation in pulse rate or body temperature, or simply by self-reporting symptoms in an app, could optimize the timing of hydrocortisone stress dosages. In theory, more insight and timely recognition of imbalances could contribute to improved quality of life and greater sense of control.

Wearable wrist devices and apps are increasingly being used in medicine, as these devices are capable of passively and continuously tracking health information (22–26), such as with the Corsano CardioWatch 287-2 (27, 28). Wearable wrist devices can stimulate a healthy lifestyle by monitoring daily activity. We hypothesized that a smart wearable device could function as the “external hypothalamus” in patients with HD, by monitoring and providing insight into (hypothalamic) imbalances, enabling more timely interventions and potentially relieve the burden on patients and/or their caregivers.

In collaboration with clinicians, patients and their representatives, and design researchers, we therefore aim to develop such a smart wearable, which can potentially be used for real-time continuous monitoring of hypothalamic imbalances in brain tumor survivors with HD. Here, we describe how the first design of this smart wearable, the *EndoWatch*, was developed with and tested by patients with acquired HD in a pilot study to explore learnability, usability, desirability as well as its barriers and facilitators.

## 2 Methods

### 2.1 Co-creating the first design of the EndoWatch

The EndoWatch research team consisted of patients, health care professionals (including pediatric oncologists and endocrinologists, dieticians, sleep experts, physiotherapists, statisticians) from the Princess Máxima Center for pediatric oncology (Utrecht, the Netherlands), adult endocrinologists and a clinical physicist from the University Medical Center Utrecht (UMCU), design engineers from Corsano Health B.V., design researchers from the Eindhoven University of Technology (TU/e), and patient representatives from the Dutch Pituitary Foundation and the Dutch Pediatric Cancer Society. Throughout the project, biweekly meetings were held with the principal investigators, and meetings involving patient representatives were held every four months.

The EndoWatch was designed as an adaptation of the Corsano CardioWatch 287-2, originally designed by Corsano Health B.V. for real-time continuous monitoring of cardio-respiratory function in adult patients. The Corsano CardioWatch 287–2 was selected as the investigational device for this project, because of its robust and certified data and research platform, and firmware that could be adapted for this purpose. The Corsano CardioWatch 287–2 is a European Union (EU) European Conformity (CE) Medical Device Regulation (MDR) and United States Food and Drug Administration (FDA) certified wearable, designed for medical use in research and clinical practice. It consists of a wearable sensor that continuously monitors and transmits health data to an application (app) via Bluetooth connection, enabling patients, health care professionals (HCPs) and researchers access to real-time data in a cloud-based research platform with best practices security, compliant to the General Data Protection Regulation (GDPR) and Health Insurance Portability and Accountability Act (HIPAA) (28–30). The CardioWatch derives vital information from multiple sensors: accelerometry (ACC), photoplethysmography (PPG), electrocardiogram (ECG), temperature and Galvanic skin response (GSR). (**Figure 2**) In this project, the intended population and use of the CardioWatch 287–2 extend beyond the original scope of its EU CE MDR and FDA certifications.

**Figure 2:**
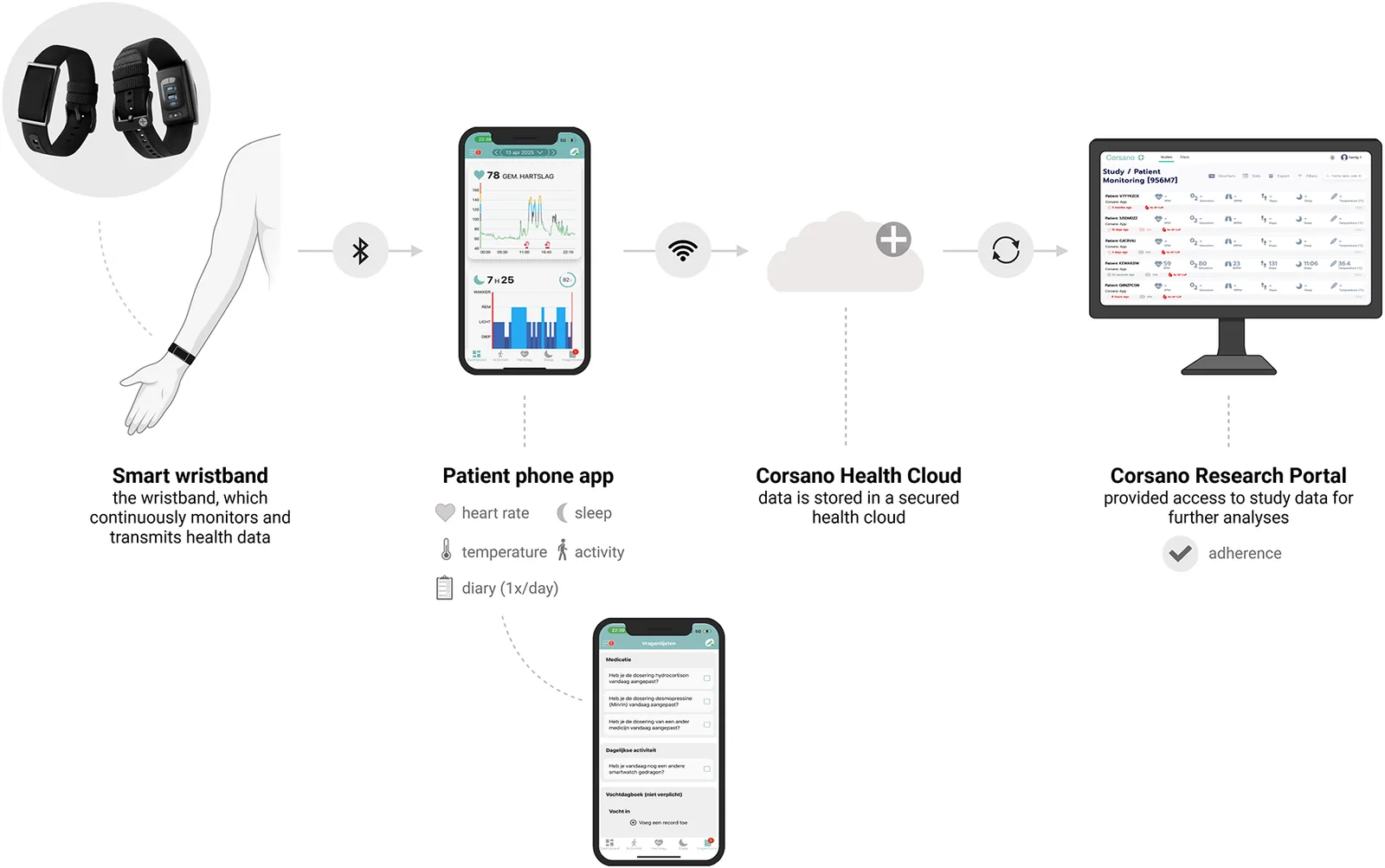
Schematic representation of the device used in the EndoWatch-I study. Created in BioRender. Hulsmann, S. (2026) https://BioRender.com/z5fsx9y.

The first phase of the development of the EndoWatch aimed to incorporate the wishes and perspectives of patients with HD and their parents into the EndoWatch design, based on their personal experience with HD. First, the EndoWatch concept, the development of a smart wearable for real-time continuous monitoring of hypothalamic imbalances in brain tumor survivors with HD, was introduced to five patient-parent dyads, to explore their overall perception of such a device. In addition, they were asked about current self-management of symptoms of hypothalamic-pituitary dysfunction, including temperature dysregulation, sleep problems, obesity, activity and timing of hydrocortisone stress dosage, and about their unmet needs. The input from these patient-parent dyads was discussed within the multidisciplinary research team and translated into specific design requirements. Patient and parent preferences were considered alongside the technical possibilities of the available Corsano monitoring platform, with particular attention to which parameters should be monitored, how information should be presented, and which functionalities should be incorporated into the app. These explorative discussions, informed the research team about the selection of health parameters that should be monitored by the EndoWatch and the information that should be made accessible through the accompanying mobile app. The idea of a patient diary to be included in the app was supported by the patient-parent dyads. The following health parameters were identified for the first design of the EndoWatch: core body temperature (CBT, in degrees Celsius), pulse rate (PR, in beats per minute), sleep stages (awake, light sleep, deep sleep, REM sleep), sleep score (in %), activity type (resting, walking, running, cycling) and number of steps per day. Additionally, a patient diary (ie, short questionnaire) was incorporated into the app to enable patients to report symptoms, episodes of physical or emotional stress, and/or changes in medication. The app interface included a dashboard that displayed an overview of all health parameters (CBT, PR, sleep and activity), more detailed sections for each parameter, and a section for the patient diary. The app was available on Android and iPhone Operating System (iOS) smartphones.

### 2.2 The EndoWatch-I study

After completing this exploratory phase with the first design of the EndoWatch, a prospective observational exploratory study (the EndoWatch-I study) was conducted in patients with HD to evaluate adherence (wearing time), learnability, usability and desirability, as well as barriers and facilitators of using the first EndoWatch design. Importantly, the clinical functionality of the device, and the device’s ability to detect clinically meaningful hypothalamic dysregulation was not assessed in this first study.

Given the rarity of hypothalamic dysfunction and the exploratory nature of this study, only 10 patients were included. No sample size calculation was performed, because the study was not designed to test formal hypotheses. Patients were recruited from the pediatric endocrinology clinic and late effects clinic at the Princess Máxima Center. Patients aged 2–40 years were eligible for inclusion if they had a history of a pediatric (supra)sellar brain tumor, diagnosed before the age of 18, and symptoms of HD confirmed by an endocrinologist. HD was defined as the presence of at least two of the following: reduced resting energy expenditure (REE) <80%, as measured with indirect calorimetry (31); hyperphagia (32), overweight or obesity [defined by international criteria according to Cole et al. (33, 34)]; disturbed circadian rhythm (defined as difficulty falling asleep, staying asleep, early morning awaking or daytime sleepiness) (35); behavioral disorders (defined as presence of obsessive compulsive symptoms, hoarding and/or periods of rage) (36); temperature regulation disorder (defined as temperatures <35–36 degrees Celsius or >37.5-38.0 degrees Celsius not induced by infection); pituitary dysfunction (defined as ≥1 dysfunctional pituitary axis) or AVP-D (as diagnosed by endocrinologist) with inadequate thirst sensation (adipsia) (5). Patients were excluded if study instructions or the app could not be understood due to a language barrier or illiteracy in patient or caregiver.

Participants and parents received instructions on using the wearable and app, which was installed on the patient’s or parent’s smartphone. The phone needed to remain near the wearable to maintain Bluetooth connectivity. Real-time data of pulse rate, temperature, sleep and activity were displayed in the app. Real-time measurements of pulse rate, temperature, sleep and activity were displayed in the app. (**Figure 2**) Participants were instructed to wear the device for 14 days and to complete the diary in the app once daily, while freely exploring the app. No additional instructions were given.

The primary outcome of this first explorative study was adherence, defined as the percentage of wearing time, based on pulse rate recordings obtained from the research portal. (**Figure 2**) Secondary outcomes included learnability, usability and desirability, and barriers and facilitators of using the wearable. Learnability, usability and desirability were assessed with an end-of-study patient- or parent-reported questionnaire, based on a feasibility questionnaire that was previously used to evaluate a pain app in children with cancer (37) and a questionnaire to assess usability of the CardioWatch in adults (30). Responses were recorded on a 5-point Likert scale (1=Completely disagree, 2=Disagree, 3=Neutral, 4=Agree, 5=Completely agree). Barriers and facilitators of using the first design of the EndoWatch were explored through short semi-structured interviews with participants and/or parent(s) at the end of the study period.

In all cases, parents completed the parent questionnaire, except for patients aged ≥18 years who were able to self-report. Patients aged ≥8 years also completed the patient questionnaire. For patients ≥18 years, but unable to self-report, parents completed the questionnaire. Interviews were conducted with the patient and parents together or with parents only when necessary.

The results from the EndoWatch-I study, reflecting patient and parent experiences of the interaction with the device, were used to identify key requirements and patients’ and parents’ needs for the device to be able to support them in the management of HD. Identified barriers and user needs were discussed with the multidisciplinary research team and design engineers from Corsano, and feasible changes were translated into specific modifications of the second EndoWatch design, including the wearable and app.

### 2.3 Data analysis

Adherence (wearing time), derived from pulse rate measurements, and questionnaire responses were analyzed quantitively.

Descriptive qualitative analysis was conducted on the answers from the open-ended questions of the semi-structured interview. Interviews were recorded for transcription. A single reviewer assessed the transcripts and identified main themes and subthemes. Subsequently, subthemes were identified as either barriers or facilitators, related to the context in the interview. These themes, barriers and facilitators were discussed within the multidisciplinary team as part of the iteration process.

### 2.4 Ethical approval

The EndoWatch-I study was approved by the regional Medical Ethics Review Committee (NedMec-METC Utrecht, approval no. NL87495.041.24) in the Netherlands. Written informed consent was obtained from all study participants.

## 3 Results

### 3.1 Results from the EndoWatch-I study

Ten patients with acquired HD were included in the EndoWatch-I study, of whom 8 (80%) were children <18 years, and 2 (20%) adults ≥18 years. (**Table 1**) One of the adult participants was diagnosed with Down’s Syndrome and requires full parental care. Two patients were wheelchair-bound, and both visually and cognitively impaired. In these 3 cases, questionnaires and interviews were completed by parents only.

**Table 1:** Participant characteristics EndoWatch I-study.

Pediatric participants (<18 years) (N = 8)	
Mean age (years (SD), range)	10.8 (3.5), 6.3-15.1
Median age at diagnosis suprasellar tumor (years (IQR))	2 (0.7-6.7)
Sex (female)	2 (25.0)
Mean BMI SDS ((SD), range)	2.0 (2.0), -1.1-5.7
*Hypothalamic-pituitary dysfunction*	
Central hypocortisolism, n/N (%)	7/8 (87.5)
AVP deficiency without adipsia, n/N (%) AVP deficiency with adipsia, n/N (%)	7/8 (87.5) 4/8 (50.0)
Hypothalamic obesity, n/N (%)	3/8 (37.5)
Hypothalamic temperature dysregulation, n/N (%)	5/8 (62.5)
Sleep disturbances, n/N (%)	7/8 (87.5)
Adult participants (≥18 years) (N = 2)	
Age (years (range))	18.9-31.0
Age at diagnosis suprasellar tumor (years (range))	8.5-13.3
Sex (female)	2 (100.0)
BMI SDS (range)	1.1-4.5
*Hypothalamic-pituitary dysfunction*	
Central hypocortisolism, n/N (%)	2/2 (100.0)
AVP deficiency without adipsia, n/N (%) AVP deficiency with adipsia, n/N (%)	2/2 (100.0) -
Hypothalamic obesity, n/N (%)	1/2 (50.0)
Hypothalamic temperature dysregulation, n/N (%)	1/2 (50.0)
Sleep disturbances, n/N (%)	1/2 (50.0)

SD(S), standard deviation (score); BMI, body mass index; IQR, interquartile range; AVP, arginine vasopressin.

#### 3.1.1 Adherence

Across the 14-day study period, the median overall adherence for all 10 patients was 99.4% (IQR 22.8). **Supplementary Figure 1**, showing variation in adherence across participants and study days. The 14-day study period was completed by 8/10 patients, while 2/10 patients discontinued early. One patient (participant 6) removed the watch after 3 days (median adherence of 94.8% (range 79.9-99.7) during these 3 days); while another other patient (participant 7) wore the watch for 8/14 days (median adherence of 32.8% (IQR 28.7) during these 8 days). Reasons for early discontinuation were wristband discomfort in participant 7 and blindness-related difficulties in the other (participant 6), leading to discomfort, lack of understanding of the device and therefore low motivation.

#### 3.1.2 Learnability, usability and desirability

In total, 4/10 patients self-completed the questionnaire, and in 8/10 cases a parent (father, mother, or both) completed it. (**Figures 3a, b**) In 3/10 cases, both patient and parent versions were completed. A parent version was completed for one adult who was unable to self-report. No questionnaire data was returned for the patient who withdrew after 3 days.

**Figure 3:**
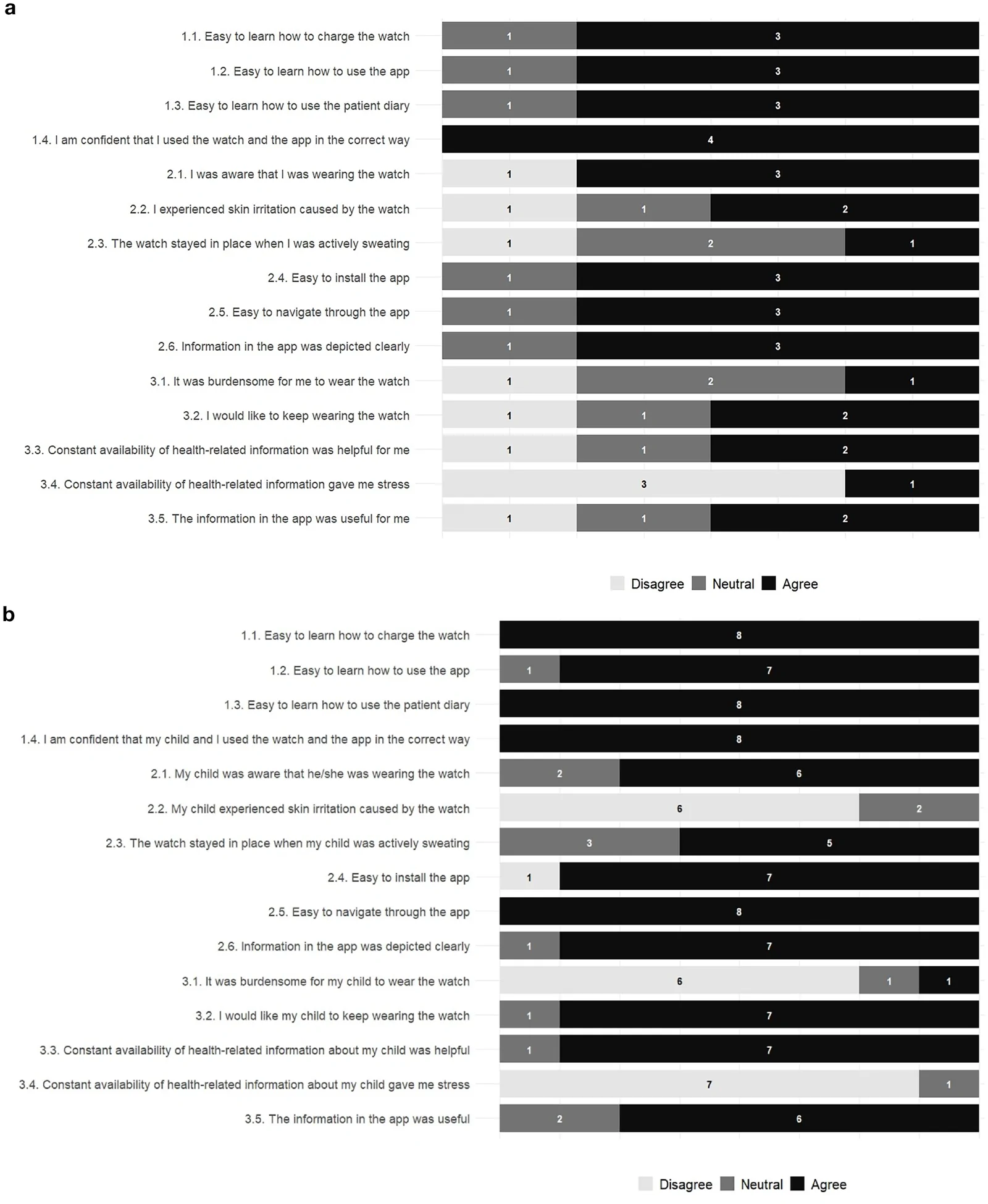
Outcomes from questionnaires completed by patients and parents. **(a)** Learnability, usability and desirability questionnaire completed by patients (N = 4). Responses rated on five-point Likert-scale divided into categories of disagree (scores 1, 2), neutral (score 3) and agree (scores 4, 5). Learnability (1.1-1.4), usability (2.1-2.6) and desirability (3.1-3.5). **(b)** Learnability, usability and desirability questionnaire completed by parents (by father, mother or by both together) (N = 8). Responses rated on five-point Likert-scale divided into categories of disagree (scores 1, 2), neutral (score 3) and agree (scores 4, 5). Learnability (1.1-1.4), usability (2.1-2.6) and desirability (3.1-3.5).

All statements on learnability of the device (1.2-1.4) were rated with a 3 (neutral), 4 (agree) or higher, by both patients and parents. For statements on usability, a distinction was made between usability of the wristband (2.1-2.3) and usability of the app (2.4-2.6). Most patients (3/4, 75%) and parents (6/8, 75%) agreed with statement 2.1; “I was aware that I was wearing the watch”/”My child was aware that he/she was wearing the watch”, though interpretation varies: either negatively (discomfort or skin irritation) or positively (active interest and participation). Skin irritation attributed to the wristband was self-reported by two patients. Skin irritation was self-reported by two patients, citing discomfort due to the synthetic strap (n=1) or inability to see the wristband due to visual impairment (n=1).

Usability of the app (statement 2.5-2.7) was overall positively rated. One parent disagreed with the statement “Easy to install the app”, due to incompatible smartphone software, though a second attempt with updated software succeeded. Most parents (6/8, 75%) did not find the watch burdensome for their child, and 7/8 (87.5%) wished for their child to continue wearing it. While most patients (3/4, 75%) and parents (7/8, 87.5%) disagreed that constant health-related information caused stress, 1/4 patients (25%) did agree.

#### 3.1.3 Barriers and facilitators

Close-out semi-structured interviews were conducted with 9/10 participants; the interview was not conducted with the participant who withdrew after 3 days. Of these interviews, 4/9 involved patients and parents together, 4/9 parents only, and 1/9 an adult patient alone. Barriers and facilitators were identified across three main themes: user friendliness of the wearable, user friendliness of the app, and technical functioning of both the wearable and app (**Table 2**).

**Table 2:** Barriers and facilitators to using the first design of the EndoWatch reported by patients and parents.

Topics	Input
	Facilitators
The design of the wearable	The absence of a screen on the wearable was perceived positively, as it reduces continuous input from data and distractions for both patient and parent. The black color of the wristband was perceived as neutral and was therefore appreciated by some participants.
User friendliness of the wearable	Attaching and detaching the wristband was easy. Charging the wearable was easy. The wearable needed to be charged only once a week.
User friendliness of the app	Navigating through the app was easy and intuitive. The layout of the app was visually appealing. The fact that all metrics were visible in the dashboard provided a useful overview. The app provides more detailed information per metric, in separate tabs. The app sent timely reminders when the wearable needed to be charged.
User friendliness of the patient diary	The patient diary was intuitive and easy to fill out. The time it took to fill out the diary was limited and therefore feasible for daily use. The app sent reminders to complete the diary if this had not yet been done.
Technical functioning of the wearable and app	The app is compatible with iOS and Android devices. The Bluetooth connection was stable when wearable and smartphone were within close proximity. When the wristband (ie, patient) and smartphone were temporarily separated, the sensor continues monitoring and reliably synchronizes all data with the app upon reconnection. The app operated smoothly without any disruptions. Notifications from the app (reminders to charge the wearable or to complete the patient diary) were received consistently.
	Barriers
The design of the wristband	The color (black) of the wristband was found to be unflattering and was not appreciated by some participants.
User friendliness of the wristband	The magnet-to-sensor contact on the wristband was not always stable during charging and was dependent on the position of the charging wire and device. The wristband resulted in slight skin irritation/itch (especially when wet after showering or sweating).
User friendliness of the app	Not all metrics were relevant for each individual participant (eg, displaying activity in steps per day is not desirable for wheelchair-bound individuals). The app could only be installed on one smartphone and required continuous Bluetooth connection with the wearable, and therefore remote monitoring for parents/caregivers was not feasible.
User friendliness of the patient diary	The diary’s questions on medication adjustments offered insufficient space to elaborate on contextual factors or specific dosage changes. The diary did not allow users to access, review or edit entries from previous days.
Technical functioning	Installing the app was not compatible with older smartphones (Apple iOS <15.0) or smartphones with limited remaining phone storage. Functions within the app did not work properly when installed on older smartphones; eg, the function of the settings menu or the patient diary.

Specific suggestions for improvement were derived from the identified barriers to use of the first EndoWatch design. One suggestion, specifically reported by parents, was to enable remote monitoring via a second app interface, allowing parents or caregivers access to patients’ real-time measurements. In the first EndoWatch design, the app could only be installed on one smartphone, which needed to be in close proximity to the wristband for Bluetooth connectivity. Another suggestion, reported by both patients and parents, was to enable access and editing of past diary entries from previous days, as this was not supported in the first EndoWatch design. Both patients and parents additionally suggested to add sections in the diary for reporting relevant contextual information alongside symptoms (eg, exceptional weather conditions explaining disturbed thermoregulation) or medication changes (eg, reason, duration, and dose of hydrocortisone increases).

Parents of patients with AVP-D and adipsia, who require fluid balance tracking to maintain optimal plasma sodium levels, recommended including a fluid diary for daily tracking of fluid intake and micturition. Lastly, patients of various ages and genders suggested offering wristbands in different colors and materials to accommodate personal preferences.

The identified user needs and barriers were translated into specific design requirements and subsequently implemented modifications in the second EndoWatch design (**Figure 4**).

**Figure 4:**
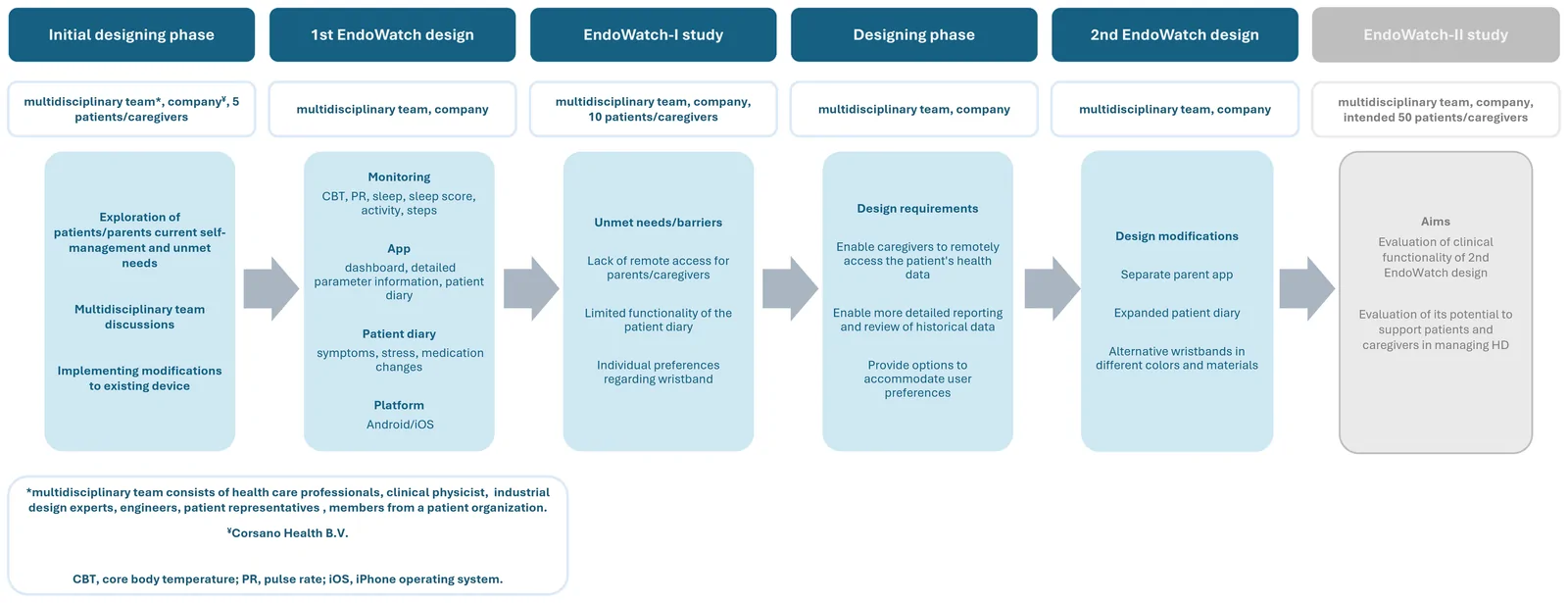
Co-creation of the EndoWatch based on translation of patient and parent feedback into design requirements and device modifications.

## 4 Discussion

Patients with HD and their parents and/or caregivers urge health care professionals for a solution to help manage the consequences of hypothalamic damage (15). The care of patients with HD places a substantial burden on patients themselves and their parents or caregivers. Caregivers currently often function as the “external hypothalamus”, striving to maintain homeostasis by preventing imbalances in thermoregulation, sleep-wake rhythm, cortisol or sodium levels, while managing behavioral problems, restricting eating behavior (in patients with hyperphagia) and promoting a healthy, physically active lifestyle to prevent rapid weight gain. Current endocrine practice is not yet making use of the development of smart wearables to provide support in the care for patients experiencing the consequences of hypothalamic-pituitary dysfunction. Here, we present the initial steps in the development of the EndoWatch, a smart wearable that may support patients with HD, their parents and caregivers. By co-creating with all relevant stakeholders in the designing process, we identified key requirements of such a device in a pilot study. In this first pilot study, we assessed adherence to the first EndoWatch design, as well as user experiences of the device. In a future study, we will test its clinical usability and its effect on management of hypothalamic imbalances, such as temperature changes.

This pilot study demonstrated that participants highly appreciate the development of the device and wish for the development of a tool to support them in daily life. We found an overall high adherence among participants. A key barrier for parents using the EndoWatch was the lack of remote access to their child’s measurements through a ‘parent version’ of the app that would facilitate remote monitoring. In this first EndoWatch design, the smartphone with the Corsano app had to remain in close proximity to the sensor for Bluetooth connection. Caregivers could therefore not view their child’s data remotely, for example, if they were at work. This was due to the fact that a Bluetooth connection was required for data synchronization between the sensor on the wearable and the smartphone with the app. A WiFi/5G connection or a second app for caregivers could enable remote monitoring and would be desirable in the future.

The variation in responses from the questionnaire and the interviews demonstrates that the user experiences of the current device are highly individual, and that patient perspectives may differ from parent/caregiver perspectives. Moreover, in line with previous research demonstrating that HD has many different clinical manifestations and requires an individualized treatment approach (2, 4, 5, 19, 38), certain features of the device may not be relevant for all patients with HD. The EndoWatch should therefore not be designed as a one-size-fits-all medical device, but rather as a system that can be personalized to meet individual needs. Co-creating the device with patients, parents, patient representatives, healthcare professionals, and engineers, while exploring and integrating individual experiences, is essential to further develop a tool that supports those seeking additional help in managing HD.

A key strength of this project is its exploratory, user-centered approach, co-creating with patients and their representatives to identify experiences and needs in a small but heterogeneous cohort, providing guidance for subsequent device development rather than relying solely on a health professional-driven perspective. The unique collaboration within the multidisciplinary EndoWatch team, including members from a patient organization, ensures that essential aspects of developing the device are addressed. Insights from patients and parents, gathered via questionnaires and interviews, alongside expertise from the multidisciplinary team, were integrated into the EndoWatch device and app design. The project also benefits from using an existing, certified medical device as a foundation, which, together with the expertise of Corsano’s design engineers, ensures a robust and safe (data) platform that can be used for further innovation.

Of course, the project also has its limitations. Although patient experiences remain the focus throughout the project, the EndoWatch has now only been tested by a limited number of patients. Moreover, although the heterogeneity of the patients may be considered a strength of the study, it also introduces a limitation when interpreting aggregate findings of user experiences in this small cohort.

In future studies, patient inclusion will be expanded, but is expected to remain limited due to the rarity of the disease. Nevertheless, the centralization of pediatric oncology care in the Netherlands, including care for children with suprasellar tumors, provides access to a relatively large cohort of patients with hypothalamic-pituitary dysfunction. This centralization facilitates the conduct of exploratory studies and supports the development of novel tools for a selected group of patients. The limited number of patients also led to the decision not to aim for any form of validation of the device at this stage of the development, as such a study would require substantially larger cohorts. If the EndoWatch renders efficacy and proves to be useful in the management of HD in future studies, validation for its purpose may then be needed to ensure certification for its intended population and use. Since European networks are in place through the European Reference Network on Rare endocrine conditions (Endo-ERN) and the European Society for Paediatric Oncology (SIOPe), international collaboration could be explored for future studies when larger patient cohorts are required. Future studies may also include patients with HD caused by different etiologies such as congenital (eg, septo-optic dysplasia), the Rapid-onset Obesity with Hypoventilation, Hypothalamic dysfunction, and Autonomic Dysregulation (ROHHAD) syndrome or head trauma (19). Importantly, this device has never been tested or validated in children or young adults before. The design engineers were, however, able to partially modify the app interface for the scope of this first study, and will further adapt it based on user experiences. This study primarily assessed adherence and user experiences related to the practical use of the wearable and app. Future steps in this project will, however, focus on the reliability of the measurements, the potential of the device to support patients with acquired HD and their caregivers, and, ultimately the validation of its measurements within this specific patient population.

Importantly, the findings from the EndoWatch-I study were not only used to evaluate the first design, but were actively translated into design requirements for the subsequent iteration. The results from the EndoWatch-I study were evaluated and the feasibility of implementing the suggested adjustments was discussed with the design engineers from Corsano, in order to overcome the currently identified barriers to using the EndoWatch. All change requests mentioned in the previous section were granted and have now been implemented in a second design of the EndoWatch. First, a separate app was introduced to enable parents to remotely monitor their child’s health parameters (eg, pulse rate, temperature) through their own smartphone. This parent app has a simpler design and does not incorporate the patient diary; thus, in this new design, the patient diary will remain exclusively available in the patient app, which is connected to the watch. Second, the patient diary was modified to allow for more detailed reporting (eg, symptoms of low cortisol levels, stress events (physical or emotional) and contextual information, and medication changes) and to enable reviewing historical diary data. Third, alternative wristbands in different colors and materials will be provided and offered upon request by patients in future EndoWatch studies.

To provide optimal support to patients with HD, other innovative tools or applications could in theory be added to future concepts of the EndoWatch. For example, continuous monitoring of sodium levels in adipsic patients with AVP-D, who currently require monitoring of fluid balance on a daily basis to prevent hyper- or hyponatremia (39, 40). Some patients require multiple weekly hospital visits for sodium checks, whereas others monitor their levels at home using finger-prick devices (41). Continuous subcutaneous monitoring of sodium levels (similar to glucose monitoring in patients with diabetes mellitus) (42) is currently not yet available (43), but could offer substantial benefit for patients with AVP-D and adipsia, who cannot rely on their thirst to guide fluid intake. In theory, data from such a sodium sensor could be integrated into the EndoWatch to consolidate all data into a single, easily accessible app that is designed to support patients with HD. The EndoWatch design could also incorporate features in the app that could support in the management of hyperphagia and hypothalamic obesity, such as the HAPPYthalamus app (44).

In conclusion, we present the first steps in the development of the EndoWatch; a smart wearable designed to act as an external hypothalamus supporting children and young adults with acquired HD and their caregivers. This first study, evaluating adherence and user experiences in a small, heterogeneous cohort, identified unmet needs, barriers and facilitators of patients after using the first EndoWatch design. This study highlights individual needs, and emphasizes that future studies should evaluate clinical functionality on an individual basis. Future steps will therefore include a planned prospective study (EndoWatch-II study) with a larger cohort, to systematically evaluate the clinical functionality of the second EndoWatch design and its potential to support patients and caregivers in managing HD.

## Data Availability

The original contributions presented in the study are included in the article/**Supplementary Material**, further inquiries can be directed to the corresponding author/s.
