# Supplementary material for: Co-creating the EndoWatch - a smart wearable device for the management of hypothalamic dysfunction in pediatric brain tumor survivors: a pilot study
Source: Front Endocrinol (Lausanne). 2026 Oct 23;17:1963615. doi: 10.3389/fendo.2026.1963615 (PMC13739600; doi:10.3389/fendo.2026.1963615)
Supplement: Supplementary file 1 [file SupplementaryFile1.docx]

| **Participant** | **Day 1** | **Day 2** | **Day 3** | **Day 4** | **Day 5** | **Day 6** | **Day 7** | **Day 8** | **Day 9** | **Day 10** | **Day 11** | **Day 12** | **Day 13** | **Day 14** |
| --- | --- | --- | --- | --- | --- | --- | --- | --- | --- | --- | --- | --- | --- | --- |
| **1** | 100,00 | 100,00 | 99,00 | 100,00 | 99,00 | 100,00 | 89,60 | 100,00 | 96,20 | 99,70 | 100,00 | 99,30 | 100,00 | 100,00 |
| **2** | 100,00 | 100,00 | 100,00 | 74,70 | 100,00 | 98,60 | 79,50 | 100,00 | 100,00 | 77,80 | 88,50 | 100,00 | 100,00 | 93,80 |
| **3** | 99,70 | 99,00 | 100,00 | 99,30 | 99,70 | 100,00 | 96,50 | 94,10 | 88,50 | 67,00 | 100,00 | 99,70 | 100,00 | 99,00 |
| **4** | 100,00 | 80,60 | 59,40 | 99,70 | 61,80 | 98,60 | 100,00 | 100,00 | 100,00 | 99,30 | 78,80 | 99,70 | 80,90 | 100,00 |
| **5** | 100,00 | 97,90 | 99,00 | 92,70 | 97,90 | 99,70 | 98,30 | 79,20 | 63,20 | 99,70 | 95,50 | 99,30 | 100,00 | 88,40 |
| **6** | 99,70 | 94,80 | 79,90 | ,00 | ,00 | ,00 | ,00 | ,00 | ,00 | ,00 | ,00 | ,00 | ,00 | ,00 |
| **7** | 17,00 | 33,30 | 46,50 | 49,30 | 32,30 | 11,10 | ,00 | 47,90 | 24,30 | ,00 | ,00 | ,00 | ,00 | ,00 |
| **8** | 97,20 | 100,00 | 100,00 | 100,00 | 100,00 | 93,10 | 65,60 | 61,50 | 100,00 | 96,90 | 99,30 | 100,00 | 99,70 | 100,00 |
| **9** | 96,20 | 100,00 | 98,30 | 96,90 | 97,60 | 100,00 | 97,90 | 90,60 | 100,00 | 99,70 | 98,30 | 87,80 | 97,60 | 100,00 |
| **10** | 100,00 | 100,00 | 100,00 | 100,00 | 98,60 | 99,00 | 97,20 | 100,00 | 99,70 | 95,10 | 98,60 | 97,60 | 100,00 | 96,50 |

**Supplementary Figure 1.** Individual adherence data across participants and study days.

Adherence was calculated as the percentage of the day that the EndoWatch was worn. Each cell represents daily adherence for an individual participant.
Green cells represent adherence of ≥50%, orange cells represent adherence of <50%, red cells represent absence of adherence data.
